# Myocardial mRNA expression of interleukin-6 and hypoxia inducible factor-1α in neonates with congenital cardiac defects

**DOI:** 10.1186/s40348-024-00187-5

**Published:** 2024-12-21

**Authors:** Nesrine Farhat, Jaime Vazquez-Jimenez, Ruth Heying, Marie-Christine Seghaye

**Affiliations:** 1 Department of Paediatric Cardiology, University Hospital, Liège, Belgium; 2 Department of Paediatric Cardiac Surgery, University Hospital, Aachen, Germany; 3 Department of Paediatric Cardiology, University Hospital, Leuven, Belgium

**Keywords:** IL-6, HIF-1α, Gene expression, Myocardium, Neonates, Congenital heart disease, Post-operative outcome

## Abstract

**Background:**

In neonates with congenital heart disease (CHD), myocardial remodelling involves activation of inflammatory pathways. The role of hypoxemia related pathways is however unknown. This study was therefore designed to investigate myocardial mRNA expression of interleukin (IL)-6 and hypoxia-inducible factor (HIF)-1α in neonates with CHD and analyse its influence on post-operative outcome.

**Results:**

14 neonates with CHD scheduled for open cardiac surgery were studied. In group 1 (*n* = 5), pre-operative transcutaneous arterial oxygen saturation (SaO_2_) was ≤ 85% and in group 2 (*n* = 9) > 85%. Expression of IL-6- and HIF-1α-mRNA was studied on right atrial biopsy by RT-PCR and corelated to post-operative (po) outcome. Group 1 patients showed higher mean arterial blood pressure (MAP) and lower glycaemia 4 h po (*p* = 0.047 and *p* = 0.021, respectively). In the whole cohort, SaO_2_ correlated negatively with MAP (Pearson R: -0.662, *p* = 0.010). mRNA coding for IL-6 and HIF-1α was detected in the myocardium of all neonates independently of age, gender, or type of CHD. IL6-mRNA expression was not influenced by pre-operative hypoxemia but was associated with higher lactate levels in early po period (Pearson R: 0,611, *p* = 0,020). HIF-1α-mRNA expression correlated negatively with pre-operative SaO_2_ (Pearson R: -0.551, *p* = 0.04) and with aspartate aminotransferase levels 4 h po (Pearson R: 0.625, *p* = 0.017).

**Conclusion:**

Our study shows that besides inflammatory pathways, hypoxemia related pathways are activated in the myocardium of neonates with CHD. Myocardial expression of both IL-6-mRNA and HIF-1α-mRNA relates to biological markers of a worse po outcome.

## Background

In infants with congenital heart disease, mechanical stress due to hemodynamic overload, hypoxemia, and increased levels of circulating cytokines induce intra-myocardial inflammatory cytokine synthesis [1–3]. In neonates with CHD, right atrial (RA) expression of cytokine-mRNA such as IL-6-mRNA is dampened by pre-operative dexamethasone administration and this is associated with less post-operative myocardial cell damage [4].

Cytokines can be either protective, damaging or both, depending on the sequence of their production and their local concentration with potential series of pathological responses, which ultimately lead to cardiomyocyte dysfunction [2, 5].

IL-6, the main cytokine released during and after cardiac surgery with pleiotropic biological activities, is an early and reliable marker of tissue damage [2].

Most neonates with severe CHD present at least a moderate degree of hypoxemia, a condition that, similarly to hemodynamic overload, induces the expression of genes involved in myocardial remodelling [4, 6].

HIFs, belonging to those genes, build a family of nuclear transcription factors regulating the adaptive response to hypoxia in terms of oxygen supply and metabolic homeostasis [6, 7]. HIF-1 is a transcriptional activation complex that initiates the transcription of a large number of target genes such as vascular endothelial growth factor, erythropoietin, heme oxygenase-1, adrenomodullin and glucose transporter-1 [6]. It is a heterodimer composed of 2 subunits, the oxygen sensitive HIF-1α, degraded or stabilised depend on oxygenation conditions, and the constitutive HIF-1β [6–8]. HIF-1α, necessary for normal embryonic development, plays a key role in angiogenesis, erythropoiesis, control of vascular tone and apoptosis [6, 8–13].

HIF-1α, activated by insulin and IGF-2 under normoxic conditions [14], is also stabilised by inflammatory cytokines such as IL-1β, TNF-α and IGF-1, responsible for endothelial lesions comparable to those caused by hypoxia [15, 16]. HIF-1α is involved in cell protection by improving mitochondrial function, decreasing oxidative stress, and activating cardioprotective signalling pathways as it has been shown in models of ischemia-reperfusion injury [17]. This might be clinically relevant in patients with CHD as a study on autopsy specimens showed higher myocardial HIF-1α expression in those with CHD than in those without CHD [7]. Furthermore, in a study on young infants with CHD undergoing cardiac surgery, we described an association between pre-operative hypoxemia and myocardial induction and stabilisation of HIF-1α, and of its target genes vascular endothelial growth factor and endothelial nitric oxide synthase [6]. However, whether HIF-1α is expressed in the myocardium of neonates with CHD and would provide myocardial protection remains unknown.

The primary objective of this this study was therefore to test the hypothesis that IL-6 as a marker of inflammation and HIF-1α as marker for the hypoxia inducible response would be expressed at mRNA-level in the myocardium of neonates scheduled for cardiac surgery. The secondary objective was to relate this expression to pre-operative parameters and post-operative outcome variables.

## Materials and methods

### Patients

After approval by the Human Ethical Committee of the Aachen University Hospital (Aachen, Germany) and informed consent of their parents, we prospectively investigated 14 neonates aged between 12 h and 26 days (mean age: 9 days) undergoing primary corrective cardiac surgery. Among them 5 (group 1) had severe hypoxemia (SaO_2_ ≤ 85%) and 9 (group 2) had normal oxygen saturation or mild hypoxemia (SaO_2_ > 85%).

Clinical and epidemiological data including type of CHD are summarized in Table 1.

**Table 1:** Clinical and epidemiological patient data

Age (days)	9.1 ± 8.4
Weight (kg)	3.49 ± 0.47
Gender (F/M)	6/8
SaO2 (%) (n)	83.75 ± 16.4 (14)
SaO2 Group 1 (%) (n)	70 ± 21.4 (5) *
SaO2 Group 2 (%) (n)	91 ± 5 (9) *
TGA (n)	6
TAPVR (n)	4
Miscellaneous (n)	4

TGA: Transposition of Great Arteries; TAPVR: Total Anomalous Pulmonary Venous Return. SaO2: Transcutaneous arterial oxygen saturation

Miscellaneous: aortic stenosis, truncus arteriosus, pulmonary sling and pulmonary atresia with ventricular septum defect (*n* = 1 each)

*P < 0.05 between groups

Results are shown as mean value ± SD

### Cardiac operation and sampling of myocardial biopsies

Conventional general anaesthesia consisted in all cases of midazolam, fentanyl sulfate and pancuronium bromide. Cefotiam hydrochloride was given as peri-operative antibiotic prophylaxis. Dexamethasone (3 mg/m^2^ body surface area) was given immediately prior to sternotomy. After starting moderate hypothermic cardiopulmonary bypass with a flow index of 2.7 L/minute/m^2^ body surface area, aorta was cross-clamped and cardiac arrest instituted by intra-aortal injection of a 4 °C cold Calafiori blood cardioplegic solution which was re-aspirated in the right atrium. A biopsy was taken from the right atrial appendage before institution of cardiopulmonary bypass. Myocardial samples taken for reverse transcriptase polymerase chain reaction were immediately snap-frozen in liquid nitrogen and stored at − 80 °C until analysis.

### Post-operative care

Standardised care was provided. Post-operative monitoring included continuous registration of heart rate and rhythm, invasive arterial blood pressure, central venous pressure, diuresis, water balance and blood gases. Inotropic support consisted of dobutamine, adrenaline and phosphodiesterase inhibitor, as required.

Oxygenation index was calculated by the ratio between partial arterial oxygen pressure and inspired oxygen fraction and served as marker for oxygenation.

Leukocyte- and platelet count, coagulation parameters, serum levels of troponin T, glycaemia, creatinine, aspartate aminotransferase, and lactate were determined at least 4- and 24 h after the operation and were controlled as often as necessary.

### Laboratory examinations

#### Reverse transcriptase-polymerase chain reaction

Total ribonucleic acid was extracted from the atrial myocardium in all patients by using the RNeasy kit (QIAGEN Inc., Hilden, Germany). Total ribonucleic acid (2 µg) was reverse transcribed to complementary deoxyribonucleic acid with specific human primers for IL-6 and HIF-1α. Target mRNA was quantified according to standard curve and normalized to levels of the mRNA coding for the house-keeping gene 18 S.

### Statistical analysis

Normality of data distribution was ascertained by the Shapiro-Wilk test.

Results are expressed by the mean value ± standard deviation.

Variance analysis between groups was performed by one-way ANOVA analysis and mean value difference between patients’ groups by the student t-test. Correlation of independent parameters was assessed by the Pearson correlation test. Pvalues < 0.05 were considered significant and *p*-values < 0.1 were considered to indicate a tendency towards significance. Data were analysed with the Statistical Package for Social Sciences (IBM SPSS Software 25).

## Results

### Clinical data and post-operative outcome

There was no significant difference in gender, age at operation and SaO_2_ between patients belonging to different CHD-groups (TGA (*n* = 6), TAPVR (*n* = 4) and miscellaneous CHD (*n* = 4)). All neonates survived after the operation.

Patients were arbitrarily grouped according to their pre-operative SaO_2_: group 1, SaO_2_ ≤ 85% (*n* = 5); group 2, SaO_2_ > 85% (*n* = 9).

Patients belonging to group 1 showed higher mean arterial blood pressure and lower glycaemia 4 h po than the others (*p* = 0.047 and *p* = 0.021, respectively).

**Fig. 1:**
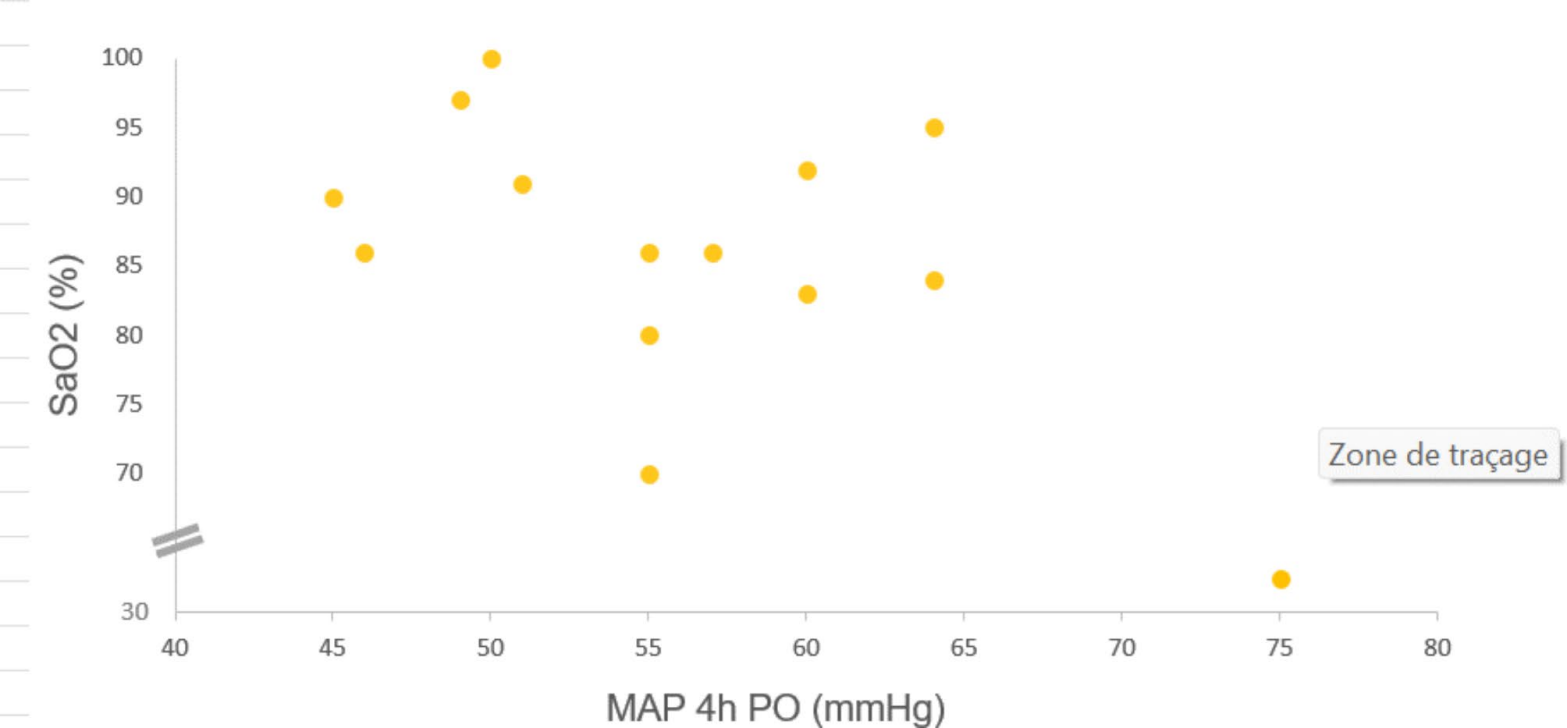
Title: Relationship between pre-operative transcutaneous arterial oxygen saturation and mean arterial blood pressure. Legend: Preoperative transcutaneous arterial oxygen saturation (SaO2) correlated with mean arterial blood pressure (MAP) 4 h post-operatively (Pearson R: -0.662, *p* = 0.010)

In the whole cohort, SaO_2_ correlated negatively with MAP (Pearson R: -0.662, *p* = 0.010) (Fig. 1) and lactate levels 4 h po (Pearson R: -0.476, *p* = 0.085) and positively with central venous pressure (Pearson R: 0.624, *p* = 0.017).

In the whole cohort, ASAT- and troponin levels 4 h po correlated with each other (Pearson R: 0.565, *p* = 0.035).

Table 2 summarises patient po parameters.

**Table 2:** Post-operative clinical and biological data according to patient groups

	Group 1 (***n*** = 5)	Group 2 (***n*** = 9)	***P***-value
MAP 4 h po (mmHg)	61.8 ± 8.3	53.0 ± 6.4	0.047
Glycaemia 4 h po (mg/dl)	114 ± 18	193 ± 64	0.021
Lactate 4 h po (mmol/l)	6.3 ± 5.2	4.94 ± 3.6	NS
Troponin T 4 h po (ng/l)	5.7 ± 2.9	5.4 ± 3.4	NS
ASAT 4 h po (UI/l)	96.8 ± 27.8	83.1 ± 27.4	NS
HIF-1α-mRNA	6.9 ± 1.8	5.1 ± 1.6	0.078
IL-6-mRNA	3.3 ± 2.1	3.3 ± 1.4	NS

MAP: mean arterial pressure; ASAT: Aspartate Amino-transferase; HIF-1α: hypoxia inducible factor-1α; IL-6: interleukin-6

Results are shown as mean value ± SD

### Laboratory investigations

IL-6 was expressed at mRNA level in the RA myocardium in all neonates included without any influence of age, gender, type of CHD or SaO_2_.

**Fig. 2:**
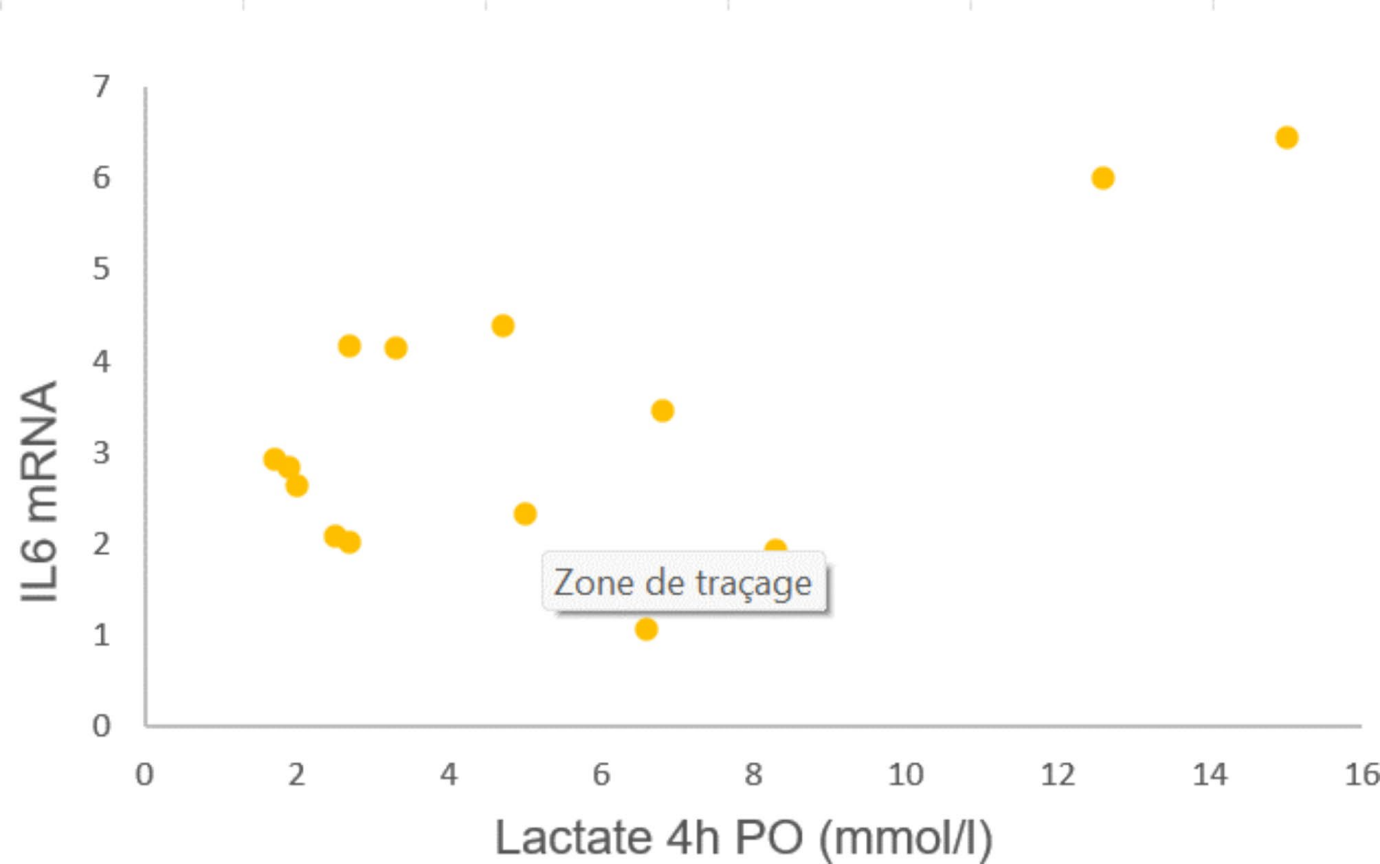
Title: Relationship between myocardial IL-6-mRNA expression and post-operative lactate levels. Legend: Myocardial IL-6-mRNA expression correlated significantly with lactate levels measured 4 h post-operatively (Pearson R: 0,611, *p* = 0,020)

IL-6-mRNA expression correlated significantly with lactate levels measured 4 h po (Pearson R: 0,611, *p* = 0,020). (Fig. 2)

Patient with IL-6-mRNA levels > Percentile 75 (*n* = 3) showed significantly higher lactate levels 4 h po than the others (*n* = 11) (*p* = 0.005).

HIF-1α was expressed at mRNA level in the RA myocardium in all neonates included without any influence of age, gender, or type of CHD, respectively.

**Fig. 3:**
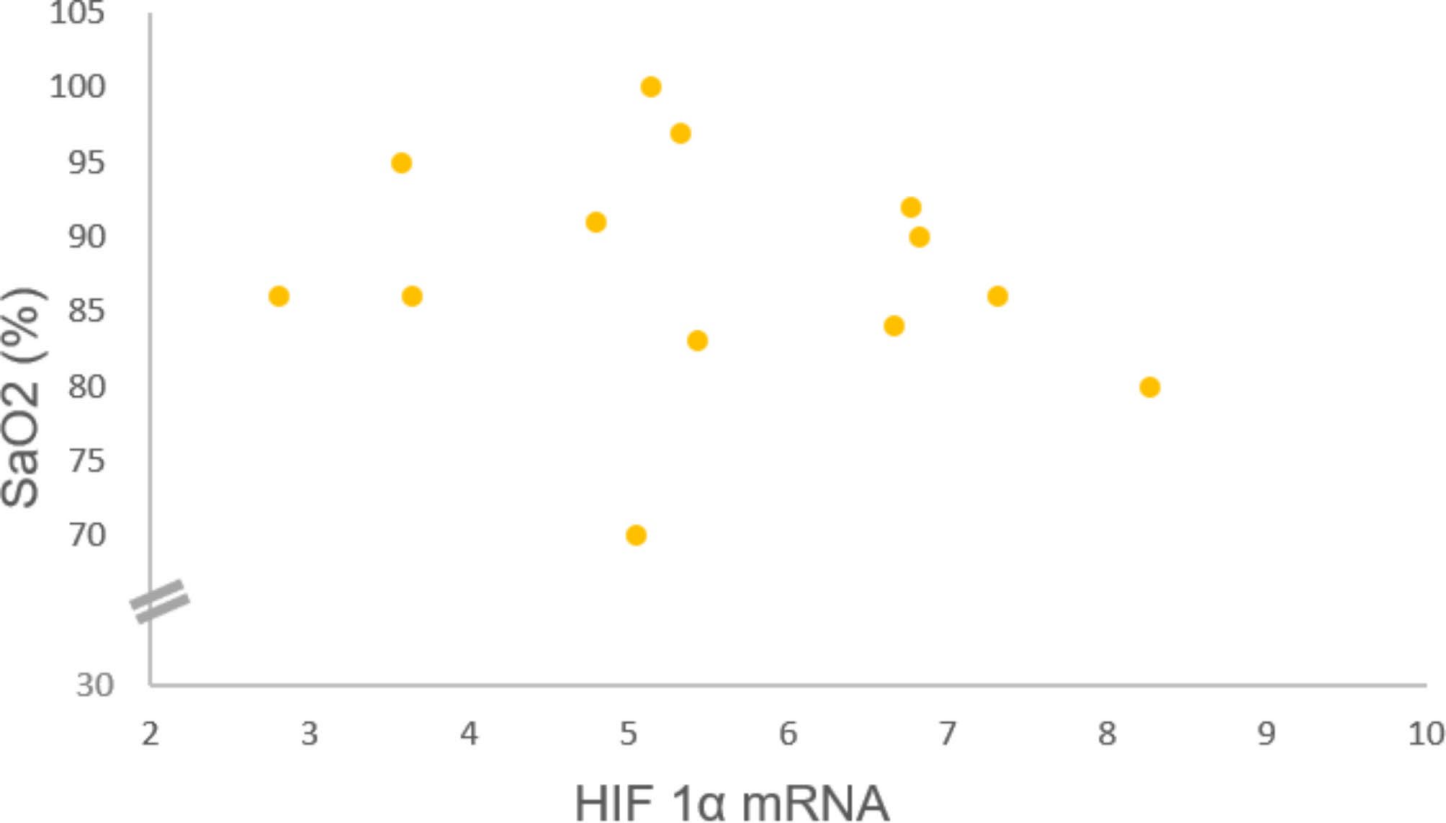
Title: Relationship between myocardial HIF-1α mRNA expression and pre-operative transcutaneous arterial oxygen saturation. Legend: Myocardial HIF-1α mRNA expression correlated with pre-operative transcutaneous oxygen saturation (SaO2) (Pearson R: -0.551, *p* = 0.04)

Myocardial expression of HIF-1α-mRNA correlated negatively with pre-operative SaO_2_ (Pearson R: -0.551, *p* = 0.04) (Fig. 3). Neonates belonging to group 1 showed higher HIF-1α-mRNA expression compared to those of group 2 (*p* = 0,078).

**Fig. 4:**
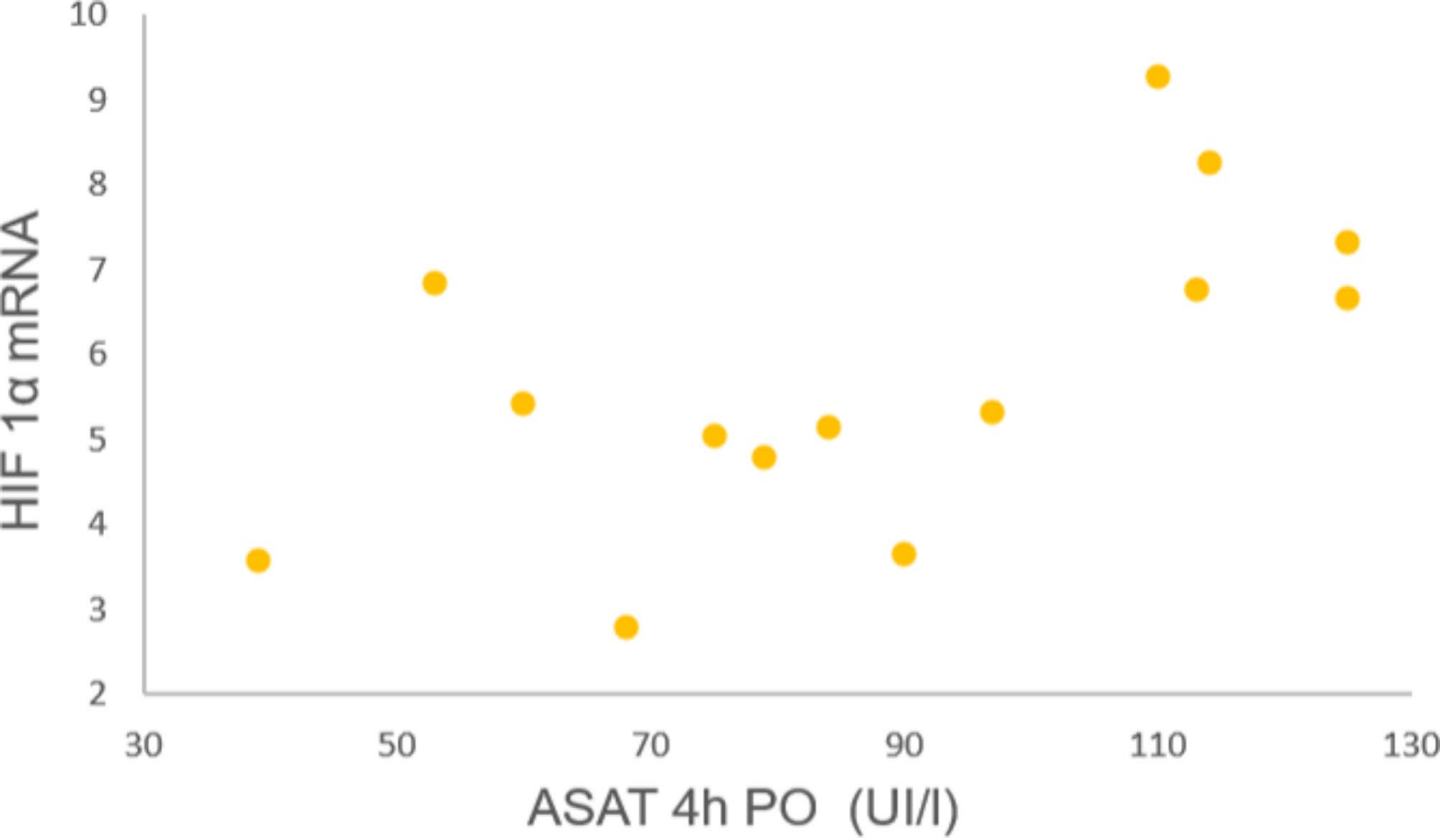
Title: Relationship between myocardial expression of HIF-1α mRNA and post-operative levels of aspartate amino-transferase. Legend: Myocardial expression of HIF-1α correlated with levels of aspartate amino-transferase (ASAT) measured 4 h post-operatively (Pearson R: 0.625, *p* = 0.017)

HIF-1α-mRNA correlated with ASAT levels 4 h po (Pearson R: 0.625, *p* = 0.017) (Fig. 4) but not with troponin levels. Patients with HIF-1α-mRNA levels > Percentile 75 (*n* = 3) showed higher ASAT levels 4 h po than the others (*n* = 11) (*p* = 0.036).

## Discussion

The primary objective of our study was to investigate whether 2 major genes involved in myocardial remodelling would be expressed in the myocardium of neonates with CHD. We confirm our previous observation on IL-6-mRNA-expression in neonatal RA myocardium [18] and show for the first time RA gene expression of HIF-1α-mRNA in this specific patient population. In this cohort, the mRNA expression of IL-6 was not influenced by pre-operative epidemiological or clinical parameters, particularly the degree of hypoxemia. This does not support the assumption that hypoxemia could activate pro-inflammatory pathways in the myocardium of neonates with CHD as it has been suggested by experimental results indicating that short intermittent hypoxia induces IL-6 production by hepatocyte derived cells independently of HIF-1 expression [19].

In the current series, we could not observe any differential IL-6-mRNA expression between the different patient groups according to their main cardiac diagnosis, most probably because of a similar RA strain in this cohort.

IL-6, involved in the pathophysiology of inflammation, auto-immunity and cancer [5, 20], plays an important role in myocardial remodelling since it promotes transforming growth factor-β mediated fibrosis [5]. In the setting of cardiac surgery, IL-6 belongs to the inflammatory mediators produced relatively early during paediatric- and neonatal cardiac surgery with cardio-pulmonary bypass support [2]. There is consistent evidence for a damaging role of IL-6 on post-operative organ function. Indeed, in neonates, circulating levels of IL-6 were correlated with the degree of myocardial cell damage and myocardial dysfunction [21]. The current results documenting a relationship between pre-operative RA myocardial mRNA-IL-6 expression and post-operative lactate blood levels suggest a depressant role of IL-6 on cardiac output lasting up to the early post-operative period and probably enhanced by the cytokine storm related to cardiac surgery [22].

Cross talk between inflammatory- and hypoxia mediated signalling pathways exists [15, 20, 23, 24]. Furthermore, HIF-1α-mRNA, expressed constitutively in many cell types, is up-regulated by hypoxia or oxidative stress via the inflammatory transcription factors NF-κB [16]. This makes the interactions between IL-6 and HIF-1α, and the members of their respective family, potentially relevant for neonates with CHD before, during and after cardiac surgery.

In this series, HIF-1α was expressed at mRNA level in all neonates irrespective of their age or diagnosis. Previous experimental data show hypoxia-related up-regulation of HIF-1α mRNA [7], which confirms our previous observations in a cohort of infants with CHD [6]. Hypoxemia induced HIF-1α-mRNA up-regulation in neonates with CHD suggests adaptation to a relative low degree of post-natal oxygen deprivation in this age group. Considering that most of the patients in this series were younger than 1 week of age and, as foetuses, were naturally subjected to chronic hypoxemia, the potential role of prenatal hypoxemia on postnatal HIF-1α-mRNA myocardial expression must be taken into account. While it is well known that HIF-1α-mRNA stability is influenced by many factors, no consistent information exists about HIF-1α-mRNA half-life in the neonatal period which is characterised by the transition from physiologic hypoxic intra-uterine to normoxic extra-uterine environment. Therefore, we cannot exclude that residual foetal HIF-1α-mRNA was present in the myocardium of some of our patients. Nevertheless, the relationship between post-natal SaO_2_ and HIF-1α-mRNA concentrations points out the role of cyanotic CHD on HIF-1α expression in human neonates.

As stated above, HIF-1α builds, with HIF-1β, the heterodimer HIF-1, the main transcription factor involved in the adaptive response to hypoxia in physiological and pathological conditions [8]. The numerous target genes of HIF-1 are involved in glucose metabolism, erythropoiesis, angiogenesis, cell survival, and increase hypoxia tolerance by allowing decreasing oxygen requirement or increasing oxygen supply [8, 13].

Given the broad range of these adaptive potential, HIF-1 provides important cardioprotective properties in ischemic heart disease and in pressure overload related heart failure [13]. However, in end-stage heart failure, HIF-1 may also participate to maladaptive metabolic reprogramming [25].

In our series, the importance of HIF-1α-mRNA expression was not associated with lower myocardial cell damage as it was not correlated with troponin T levels measured post-operatively. In contrast, the correlation between HIF-1α-mRNA and post-operative ASAT concentrations as a marker for cytolysis might indicate HIF-1α-induced activation of monocytes and neutrophils in the setting of the systemic inflammatory reaction related to paediatric cardiac surgery [16].

Besides physiological and pathological adaptation to hypoxia in post-natal life, HIF-1 plays a central role in normal embryonic development and during foetal life [9, 12, 26]. Intra-uterine hypoxia (local oxygen concentrations < 2%) is an important trigger for embryonic and foetal development, specifically for cardiogenesis [9–12]. In the embryo, HIF-1α is expressed in the myocardium depending on the degree of hypoxia as shown in the atria, ventricles, and atrio-ventricular cushions. Experiments done on animals lacking HIF-1α expression show myocardial hypoplasia and reduced myocardial trabecularisation [9, 12, 26]. HIF-1 has been shown to be involved in outflow tract remodelling that allows normal connections between both ventricles and their respective great artery and the establishment of the normal coronary vasculature [10–12].

In our series, RA HIF-1α-mRNA was not differentially expressed in the different patient groups related to their principal cardiac disease. Owing to the small patient number investigated in this series and the effect of post-natal hypoxemia in most of the neonates, the question of whether HIF-1α had any influence on the type of CHD can hardly be answered here. The post-operative clinical outcome of the neonates investigated was associated with the presence of pre-operative hypoxemia. Indeed, neonates with hypoxemia had higher early post-operative blood pressure and lower glycaemia. At this stage, an effect on vascular resistances mediated by HIF-1α cannot be ascertained but only suggested by the fact that endothelin-1, its receptors and α1-adrenergic receptors have been reported to be increased in response to hypoxia in arterial vascular smooth muscle cells via HIF-1 [25]. The relationship observed between HIF-1α-mRNA expression and early post-operative glycaemia might be interpreted as the resulting effect of hypoxia on increased insulin secretion in neonates as it has been recently shown in rodents [27].

Besides the established modulators of HIF-1α expression, constitutive variations due to different HIF-1α polymorphisms exist in humans. Single nucleotide polymorphisms in the HIF-1α gene are linked to either improved or impaired disease progression and varying adaptive responses to physiological stress, depending on the polymorphism type [28]. Few data are available in children with CHD, but previous studies suggest a link between certain EGLN1 gene polymorphisms, which code for prolyl-4-hydroxylase2 of HIF-1α, and hypoxia responses, particularly the development of collateral vessels [29].

While the patient population studied here is original and homogenous in terms of age and pre-operative condition, the patient number is small. The work was restricted to the analysis of the expression of mediators of myocardial remodelling at mRNA level. This is limiting as the biological activity of IL-6 and HIF-1α is not strictly depending on the gene expression at mRNA level. However, our previous studies in older infants showed concordance between mRNA-expression, levels- and activity of the target proteins [6].

## Conclusions

In this study, we showed that IL-6 as a marker of inflammation and HIF-1α as a marker of the hypoxia inducible response are expressed at mRNA level in the RA myocardium of neonates with CHD.

A higher IL-6 mRNA expression was associated with signs of lower cardiac output early post-operatively but was not influenced by pre-operative hypoxemia.

On the contrary, a higher HIF-1α expression was related to a higher degree of preoperative hypoxemia and with higher early post-operative cytolysis.

## Data Availability

No datasets were generated or analysed during the current study.

## Abbreviations

CHD: Congenital heart disease
mRNA: Messenger ribonucleic acid
IL6: Interleukin 6
HIF: Hypoxia-inducible factor
SaO_2_: Transcutaneous arterial oxygen saturation
MAP: Mean arterial blood pressure
RA: Right atrial
TGA: Transposition of great arteries
TAPVR: Total anomalous pulmonary venous
ASAT: Aspartate amino-transferase
RV: Right ventricular
